# How to train health personnel to protect themselves from SARS-CoV-2 (novel coronavirus) infection when caring for a patient or suspected case

**DOI:** 10.3352/jeehp.2020.17.10

**Published:** 2020-03-07

**Authors:** Sun Huh

**Affiliations:** Department of Parasitology and Institute of Medical Education, College of Medicine, Hallym University, Chuncheon, Korea; The Catholic University of Korea, Korea

In Korea, the first case of coronavirus disease 2019 (COVID-19; the causative agent of which is known as severe acute respiratory syndrome coronavirus 2 [SARS-CoV-2], previously referred to as the novel coronavirus) was detected on January 21, 2020 [1], after which the number of positive cases steeply increased to 6,284 on March 6, 2020 [2]. Furthermore, the number of deaths in Korea due to this infection was 42 as of March 6, 2020 (Fig. 1).

Starting on February 17, 2020, the number of positive cases began to increase dramatically, originating from a cluster of infections in Daegu. The number of positive cases in Daegu reached 4,693 (74.7% of the total in Korea) on March 6, 2020. In the context of the COVID-19 epidemic, this editorial aims to describe the safety actions that health personnel in Korea must take to protect themselves from this viral infection. This information may also be useful for training health personnel who are coping with COVID-19 to take steps to maximize their safety.

In Daegu, the medical institutes were not able to admit all the positive cases. Therefore, some infected persons were isolated in their homes. Nonetheless, the Korean government will secure clinical bed facilities for them in the near future. If all persons who test positive for COVID-19 are hospitalized, they will be able to receive the appropriate medical services. All costs will be covered by the Korean government through the Prevention of Contagious Diseases Act [3]. To ensure the provision of clinical beds for all infected persons, the Korean government began to use various accommodations as hospitalization facilities, including training institutes of public organizations or private companies with the owners’ permission. More than 2,000 infected persons with no symptoms or mild symptoms will be hospitalized in the new facilities.

In addition to accommodations, health personnel is needed to care for COVID-19 patients. The safety of health personnel is essential in order for them to provide the best possible medical services for infected persons. Since the dramatic spike in cases in Daegu, some nurses at hospitals in Daegu have worked for 12 hours a day in 2 shifts. Before the present viral epidemic, they worked for 8 hours a day in 3 shifts. Furthermore, physicians and other health professionals are also working harder due to the shortage of health professionals to care for patients. In addition to the increased workload, health professionals now face the possibility of being exposed to and infected with SARS-CoV-2.

Health personnel should be protected from this viral infection through appropriate adherence to the relevant guidelines. Three major tips for medical institutes on the Korea Centers for Disease Control and Prevention (KCDC) homepage were posted on January 29, 2020, as follows:

*Behavioral tips for the prevention of novel coronavirus infection by medical institutes* – January 29, 2020, KCDC [4]
1. Wear a mask or other protective device when caring for patients with respiratory diseases
2. Check the international travel history of patients, including countries where there is a coronavirus epidemic.
3. If a novel coronavirus infection is suspected, report the patient to the health center of the local jurisdiction.

Of these 3 tips, the first one describes safety measures to be taken by health personnel.

The KCDC has repeatedly announced the following 6 tips for members of the general public to protect themselves from contracting COVID-19 [4]:

*Behavioral tips for the public for the prevention of* COVID-19
1. Wash your hands often and thoroughly with soap and running water for 30 seconds or longer.
If soap and water are not available, use an alcohol-based hand sanitizer.
2. Please follow appropriate coughing etiquette if you have a cough or any respiratory symptoms.
If you do not have a mask, cover your mouth and nose with your sleeve when coughing.
If you cover your mouth and nose with a tissue, throw it away and wash your hands.
3. Do not touch your eyes, nose, and mouth with your hands.
4. Wear a mask when visiting medical facilities.
5. Do not visit crowded places.
6. Do not come into close contact with people who have symptoms such as fever or cough.

Of the 6 tips listed above, the final item is not applicable for health personnel because they must work in close proximity to patients or suspected cases. The other 5 tips apply to health personnel, as well as to the general public.

The protective devices mentioned above are presented in Table 1 [5,6], which is modified from the World Health Organization novel coronavirus (COVID-19) v3 operational support & logistics disease commodity packages, 2020. 2. 7. [7].

## Appropriate wearing and removing personal protective equipment

Prepare the items according to the recommended range of personal protective equipment in advance and wear them in the proper order and manner as follows:

1. Hair should be neatly tied or fixed and watch and jewelry should be removed to prevent contamination.
2. Health personnel should drink water before wearing protective equipment to prevent dehydration. After that, he or she should go to the bathroom before work.
3. If there found contamination or damage after wearing, personal protective equipment should be replaced.
4. Health personnel should be shifted if the gloves are wet.

The guideline for the care of severe patients with novel coronavirus (COVID-19) infection published by the Korean Society of Critical Care Medicine, the Korean Academy of Tuberculosis and Respiratory Diseases, the Korean Society of Infectious Diseases, and the Korean Society for Antimicrobial Chemotherapy contains the following guidance [8]:

l. Health personnel should wear basic personal protective equipment, including a Korea filter (KF)94 respirator, goggles, face protector, disposable waterproof long-arm gown, and gloves.
2. When performing tracheal intubation, laryngoscopy, and cardiopulmonary resuscitation, level D personal protective equipment is essential, including a KF94 respirator, goggles, or face protector, whole-body protective clothing, gloves, cap, or hood.
3. It is recommended that at least 1 physician and 1 assistant nurse wear a powered air-purifying respirator if available.

The Korean government ordered 742 public health physicians in training to care for patients with COVID-19 on March 5, 2020. Public health physicians are male graduates of medical schools who must fulfill their national defense duty by serving for 3 years in a medically vulnerable area. They received the following training: diagnostic testing for COVID-19, responses to infectious diseases, how to wear protective clothing, and management of novel infectious diseases in screening clinics [9]. Furthermore, some hospitals have dispatched physicians to serve at hospitals in the epidemic area, and some physicians have voluntarily participated in patient care in epidemic areas.

Is it inevitable that health personnel will be infected with SARS-CoV-2 when caring for patients? It is certainly the case that in Korea, physicians and nurses have been infected with SARS-CoV-2 while providing care. At a single institute, more than 10 physicians or nurses were reported to have contracted COVID-19 while providing patient care [10]. Emergency medical technicians have also been infected with the virus while transporting patients.

The answer to this problem is simple: follow the tip to “wear a mask or other protective device when caring the patients with respiratory diseases.” Various types of personal protective equipment are described in Table 1. For the safety of health personnel from droplet-transmitted infections, continuous National Institute for Occupational Safety and Health-approved (N95) respirator use showed superior protection than targeted N95 respirator use, surgical mask use, or usual mask-wearing practices [11]. In a set of personal protection guidelines for health personnel responding to the COVID-19 epidemic published by Chinese physicians, it was recommended that personal protective equipment of various levels should be used as appropriate in multiple distinct zones. Those guidelines also indicated the specific devices that health personnel should use [12]. Therefore, ensuring an adequate supply of these devices is essential for the safety of health personnel. Antiseptic or safety techniques are dealt with in undergraduate coursework at medical schools and schools for other health professionals. For example, training on reducing the rates of needlestick or sharp injuries is necessary [13]. If health personnel follow the principles of safety and antiseptic techniques, the risk of nosocomial viral infections from patients will be lowered. For example, the methods to collect the specimen from the nasopharynx, oropharynx, and sputum were presented in Supplement 1.

Health personnel responding to the COVID-19 epidemic face other issues in addition to nosocomial infection, including physical workload and psychological stress. The high physical workload is difficult to alleviate due to the shortage of health personnel able to participate in the urgent response to the epidemic. The only solutions may be regular exercise, stretching, and balanced meals. The psychological stress experienced by health personnel may not yet be visible. Some personnel do not leave the hospital in order to have time to rest or due to their fear of potentially transmitting the infection to their family members if they become infected. Regular testing of health personnel for COVID-19 is required, even when they have no symptoms or signs of the disease. When health personnel have the time to rest during patient care, psychological support should be provided for them. Furthermore, to relieve their physical and psychological stress, ongoing communication and mutual encouragement among health personnel should be encouraged.

Eczema on the face is another frequent complication of long-term use of personal protective equipment, including goggles and a respirator. To relieve these symptoms, medicated creams or ointments may be used. Placing an adhesive bandage on the skin is a preventive method.

Existing guidelines for the safety of health personnel during a sudden epidemic of viral infectious disease do not contain thorough documentation of aspects of safety other than personal protective equipment. In addition to preventing nosocomial infections, the physical safety and psychological stability of health personnel should be considered. Therefore, more precise guidelines or training materials should be integrated into the undergraduate curriculum of health professions and the training of health personnel at medical institutes.

## Figures and Tables

**Fig. 1.**
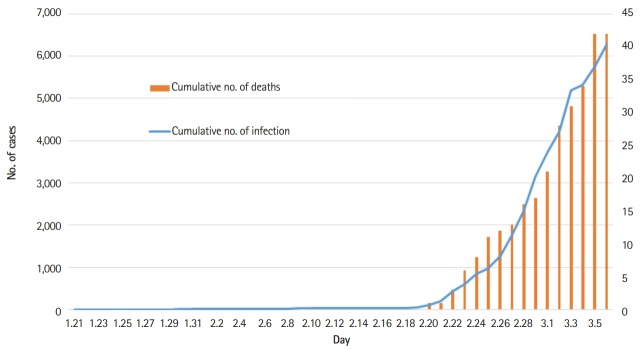
The cumulative number of SARS-CoV-2 infections and the number of deaths from January 21 to March 6, 2020 in the Republic of Korea.

**Table 1.** Recommendations for personal protective equipment to be used by health personnel to prevent SARS-CoV-2 infection

Situation or behavior	Respiratory protection			Whole body protection			Eye protection
	Surgical mask	Respirator protector equivalent to N95 or equal level	Powered air-purifying respirator	Disposable glove^a)^	Disposable long-arm gown	Whole body protective clothing including slippers	Safety glasses (or face shield)
Quarantine (epidemiological investigation)		R		R		R	R
Screening desk		R		R	R		
Reception and guide for quarantine clinics		R		R	R		
Treatment and nursing care in quarantine clinics		R		R	R	R	R
Transport (ambulance driver)^b)^		R		R			
Transport (quarantine officer, health center officer, and emergency medical technicians)		R		R		R	R
Disinfection of ambulance		R		R		R	R
Visiting, treatment, and nursing care for suspected case		R		R	R	R	R
Processes that produce aerosols^c)^		R	R	R	R	R	R
Radiological exams		R	R	R	R	R	R
Respiratory sampling		R	R	R	R	R	R
Sample handling (laboratory)^d)^		R	R	R	R	R	R
Transport of well-packaged specimens				R			
Transport of cadavers		R		R		R	
Cleaning and disinfection of hospital rooms		R		R	R	R	R
Packaging and handling of medical waste		R		R	R	R	R
Transport of medical waste	R			R	R		

R, recommended.

a) Double gloves should be worn considering the risk of tear of gloves or risk of exposure to infections when conducting medical treatment, nursing care, testing, and cleaning of suspected and confirmed patient areas.

b) If the driver’s seat is not shielded or if there is a chance of contact with a suspected or confirmed patient, wear whole body protective clothing, including shoes, KF94 equivalent respirator, and gloves (add safety glasses or face shield if necessary).

c) Processes that produce aerosols include endotracheal intubation, cardiopulmonary resuscitation, bronchoscopy, airway aspiration, tracheostomy care, necropsy, continuous positive air pressure, nebulizer therapy, and induction of sputum discharge.

d) The selection, use, and management of personal protective equipment in specimen handling laboratories shall be following the Laboratory Biosafety Guidelines (Biological Safety Evaluation Division, National Institute of Health, Korea Centers for Diseases Control and Prevention). Wearing a long-arm gown and disposable glove is required to work in the Biosafety desk of class II level [6].

## Supplementary materials

Data files are available from https://doi.org/10.7910/DVN/3KZYW6

**Supplement 1.** Video file for methods to collect the specimen from the nasopharynx, oropharynx, and sputum (The video was photographed by Dr. Seung Soon Lee, Hallym University on March 9, 2020. Informed consent was obtained from the examinee).
